# Organization of some repetitive DNAs and B chromosomes in the grasshopper *Eumastusia koebelei koebelei* (Rehn, 1909) (Orthoptera, Acrididae, Leptysminae)

**DOI:** 10.3897/CompCytogen.v10i2.7609

**Published:** 2016-04-06

**Authors:** Allison Anjos, Vilma Loreto, Diogo C. Cabral-de-Mello

**Affiliations:** 1 Departamento de Biologia, Instituto de Biociências, UNESP, Rio Claro, São Paulo, CEP 13506-900, Brazil; 2 Departamento de Genética, Centro de Ciências Biológicas, UFPE, Recife, Pernambuco, 50732-970, Brazil

**Keywords:** Cytogenetics, DOP-PCR, FISH, multigene family, Orthoptera

## Abstract

B chromosomes occur in approximately 15% of eukaryotes and are usually heterochromatic and rich in repetitive DNAs. Here we describe characteristics of a B chromosome in the grasshopper *Eumastusia koebelei koebelei* (Rehn, 1909) through classical cytogenetic methods and mapping of some repetitive DNAs, including multigene families, telomeric repeats and a DNA fraction enriched with repetitive DNAs obtained from DOP-PCR. *Eumastusia koebelei koebelei* presented 2n=23, X0 and, in one individual, two copies of the same variant of a B chromosome were noticed, which are associated during meiosis. The C-positive blocks were located in the pericentromeric regions of the standard complement and along the entire length of the B chromosomes. Some G+C-rich heterochromatic blocks were noticed, including conspicuous blocks in the B chromosomes. The mapping of 18S rDNA and U2 snDNA revealed only autosomal clusters, and the telomeric probe hybridized in terminal regions. Finally, the DOP-PCR probe obtained from an individual without a B chromosome revealed signals in the heterochromatic regions, including the entire length of the B chromosome. The possible intraspecific origin of the B chromosomes, due to the shared pool of repetitive DNAs between the A and B chromosomes and the possible consequences of their association are discussed.

## Introduction

The grasshoppers of the subfamily Leptysminae (Orthoptera, Acrididae) are divided into two tribes, Leptysmini and Tetrataeniini, comprising 75 species distributed exclusively in the Neotropical region (Amedegnato 1974, Carbonell 1977, Roberts and Carbonell 1982). The genus *Eumastusia* (Rehn, 1909) belongs to Tetrataeniini, with one species and two subspecies recognized, *Eumastusia koebelei koebelei* (Rehn, 1909) and *Eumastusia koebelei chapadendis* Roberts & Carbonell, 1980. For Leptysminae, few chromosomal data are available and, as in other Acrididae grasshoppers, most species exhibit the basic karyotype 2n=23, X0♂ with acrotelocentric chromosomes (Mesa et al. 1982, Loreto and de Souza 2000, Rocha et al. 2004). However, derived karyotypes arising from diploid number reduction were reported in *Stenopola pallida* (Bruner, 1906), *Leptysma argentina* Bruner, 1906 and *Tetrataenia surinama* (Linnaeus, 1764) (Mesa et al. 1982, Bidau and Hasson 1984). Additionally, B chromosomes have been reported in some species (Bidau and Hasson 1984, Confalonieri and Bidau 1986, Rocha et al. 2004), but no studies using molecular cytogenetic approaches have been conducted to elucidate the origin and evolution of these chromosomes.

B chromosomes are present in approximately 15% of eukaryote species and although discovered in 1907, they remain a mystery regarding their origin and evolution in most species (Houben et al. 2014). They are dispensable elements, largely known for their selfish nature as genomic parasites with patterns of non-Mendelian inheritance and a tendency to accumulate (Camacho 2005, Houben et al. 2014). These elements may arise from chromosomes of the carrier species or as a result of interspecific hybridization (Camacho et al. 2000), and they have their own evolutionary fate in different species and types of B chromosome (Banaei-Moghaddam et al. 2015). In some species, iso B chromosomes, formed by two identical arms, were described, which usually arise from centromere misdivision of telo- or acrocentric B chromosomes (see for example Grieco and Bidau 2000, Marques et al. 2012, Valente et al. 2014).

The accumulation of repetitive DNAs as an evolutionary process has been frequently reported for B chromosomes (Camacho 2005, Houben et al. 2014, Banaei-Moghaddam et al. 2015). These repetitive DNAs have been informative for understanding chromosomal and genomic evolution among grasshoppers (Cabrero and Camacho 2008, Cabrero et al. 2009, Cabral-de-Mello et al. 2011a, Anjos et al. 2015, Camacho et al. 2015, Palacios-Gimenez et al. 2015), as well as the possible evolutionary history of B chromosomes (Teruel et al. 2010, Oliveira et al. 2010, Bueno et al. 2013). To contribute to the understanding of chromosomal diversification, B chromosome evolution and patterns of repetitive DNA organization in Leptysminae, a poorly studied group, we analyzed the chromosomes of the species *Eumastusia koebelei koebelei* (Acrididae, Leptysminae). The analyses were performed through conventional and differential chromosome staining and through fluorescent *in situ* hybridization (FISH) using distinct probes, such as 18S rDNA, the TTAGG telomeric motif, U2 snDNA and a repetitive DNA fraction obtained by degenerate oligonucleotide-primed PCR (DOP-PCR).

## Material and methods

Ten adult males of *Eumastusia koebelei koebelei* were collected in Serrolândia/Pernambuco, Brazil. The testes were fixed in Carnoy’s solution (3:1 absolute ethanol:acetic acid) and stored at -20°C until use. For chromosomal preparations, the tissues were macerated in a drop of 50% acetic acid and the slides were dried using a hot plate at 40–45°C. All individuals were studied using conventional staining with 5% Giemsa to describe the general karyotype structure. C-banding was performed according to Sumner (1972) and fluorochrome staining (CMA_3_/DA/DAPI) was performed according to Schweizer et al. (1983).

The 18S ribosomal DNA (rDNA) sequence and the U2 snDNA were obtained through polymerase chain reaction (PCR) from the genomes of *Dichotomius semisquamosus* (Curtis, 1845) (Coleoptera, Scarabaeidae) and *Abracris flavolineata* (De Geer, 1773) (Orthoptera, Acrididae), respectively, using primers described by Cabral-de-Mello et al. (2010) and Bueno et al. (2013). Telomeric probes were obtained by PCR using the complementary primers (TTAGG)_5_ and (CCTAA)_5_, following the protocol proposed by Ijdo et al. (1991). Genomic amplification preferential for the repetitive DNAs was performed through DOP-PCR using as template the DNA from an individual without B chromosomes (Telenius et al. 1992). The DOP primer (5’ CCG ACT CGA GNN NNN NAT GTG G3’) was used following the specifications described by Mazzuchelli and Martins (2009).

The 18S rDNA probe and DOP-PCR product were labeled using biotin-14-dATP through nick translation (Invitrogen, San Diego, CA, USA), while the telomeric probe and U2 snDNA were labeled through PCR with digoxigenin-11-dUTP (Roche, Mannheim, Germany). Fluorescent *in situ* hybridization (FISH) was performed according to the protocol proposed by Pinkel et al. (1986) with modifications described by Cabral-de-Mello et al. (2010). Single or double-color FISH was performed with the distinct probes and at least 200 ng of each probe was used. Probes labeled with biotin-14-dATP were detected using streptavidin-Alexa Fluor 488 (Invitrogen), and probes labeled with digoxigenin-11-dUTP were detected using anti-digoxigenin-Rhodamine (Roche). All preparations were counterstained with 4’,6-diamidino-2-phenylindole (DAPI) and mounted in Vectashield (Vector, Burlingame, CA, USA). Chromosomes and signals were observed using an Olympus BX61 epifluorescence microscope equipped with appropriate filters. Photographs were recorded with a DP70 cooled digital camera. The images were merged and optimized for brightness and contrast with Adobe Photoshop CS2.

## Results and discussion

**Figure 1.**
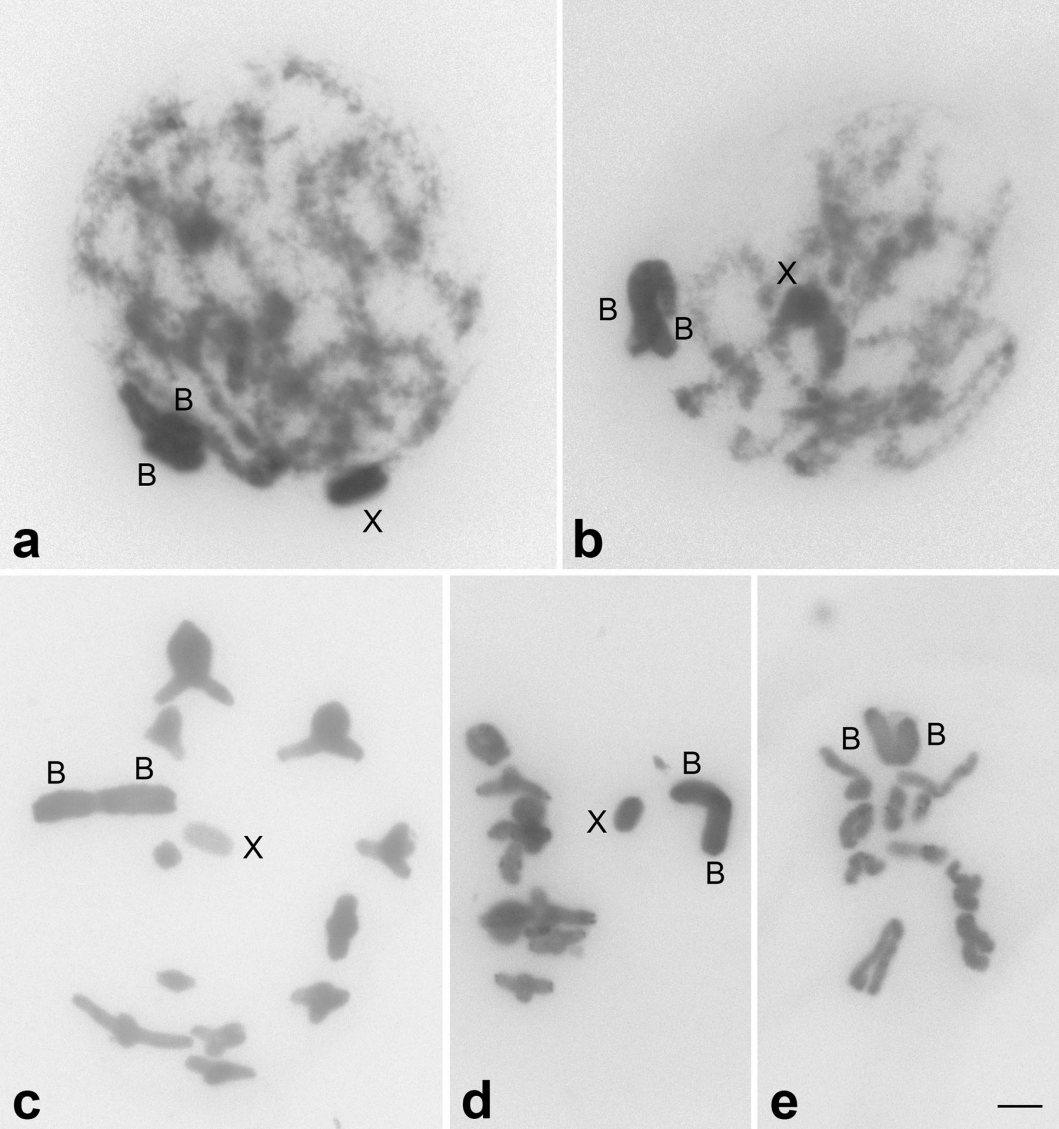
Conventional staining with Giemsa in meiotic cells of *Eumastusia koebelei koebelei* harboring B chromosomes. **a** zygotene **b** early pachytene **c** metaphase I **d** anaphase I **e** metaphase II. B chromosomes are associated side by side in initial meiosis (**a, b**) and by centromere in other cells (**c–e**). These chromosomes are also segregated to the same pole in **d** and maintained together in metaphase II (**e**). X and B chromosomes are indicated. Bar: 5 µm.

The karyotype of *Eumastusia koebelei koebelei* is in accordance with previous descriptions (Mesa and Fontanetti 1983), corresponding to the modal karyotype for grasshoppers (Hewitt 1979, Mesa et al. 1982), which consists of 23 acrotelocentric chromosomes and the X0 sex-determining system in males (Figure 1). This chromosomal pattern is also frequent in Leptysminae, occurring in 20 of 22 species studied (Mesa et al. 1982, Bidau and Hasson 1984, Confalonieri and Bidau 1986, Loreto and de Souza 2000, Rocha et al. 2004). Among the ten analyzed individuals, one carried two acrocentric B chromosomes, which showed differential or similar condensation between them, depending on the cell analyzed (Figure 1). For the other Leptysminae, distinct variants of B chromosomes were previously observed in *Stenopola dorsalis* (Thunberg, 1827) (Rocha et al. 2004), *Cylindrotettix obscurus* (Thunberg, 1827) and *Cylindrotettix santarosae* Roberts, 1975 (Confalonieri and Bidau 1986). Throughout meiosis, the two B chromosomes were associated, including metaphase II (Figure 1). From initial meiosis to pachytene, the B chromosomes were associated side by side, apparently linked by the centromere (Figure 1a, b). After diplotene, these elements remained connected by centromeres (Figure 1c–e), appearing as a single large biarmed chromosome under conventional analysis. These two B chromosomes segregate to the same pole during anaphase I (Figure 1d). This association suggests similarity between the two B chromosomes and that they could be two copies of the same B variant. Moreover, this association could influence the inheritance of these extra chromosomes, increasing the possibility of their segregation to the same anaphase pole, causing accumulation of these elements. In other grasshoppers, there are examples of acrocentric B chromosomes that are not associated throughout meiosis, such as in *Rhammatocerus brasiliensis* (Bruner, 1904) (Loreto et al. 2008).

**Figure 2.**
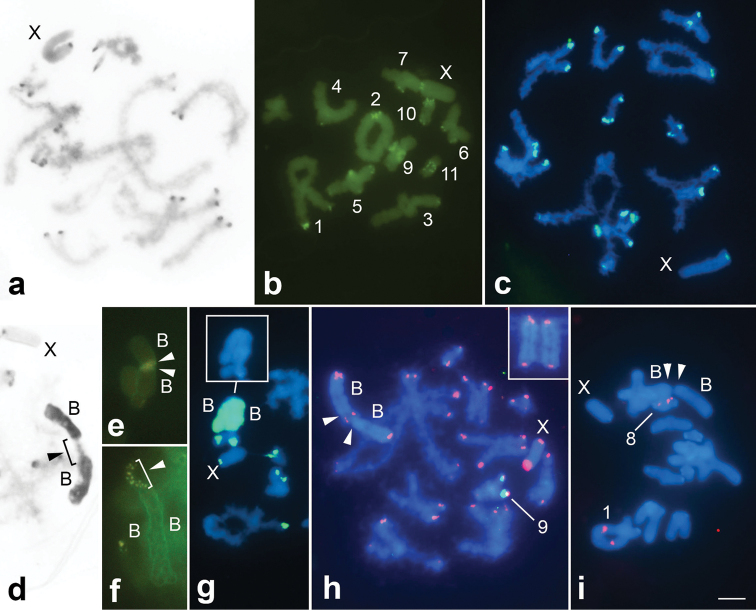
C-banding (**a, d**), CMA_3_ staining (**b, e, f**) and FISH using as probes DOP-PCR (**c, g**), 18S rDNA-green and TTAGG-red (**h**) and U2 snDNA (**i**) in meiotic cells of *Eumastusia koebelei koebelei* with B chromosomes (**d–i**) and without them (**a–c**). **a, h** late pachytene **b** early diakinesis **c, d, g** diplotene **e, i** metaphase I **f** zygotene. Images **d–g** partially highlight B chromosomes. X, B and other chromosomes harboring specific signals are indicated; arrowheads point to the centromeres of B chromosomes. Inserts in **g**, **h** highlight B chromosomes. Bar: 5 µm.

C-banding revealed pericentromeric C-positive heterochromatic blocks in the A complement (Figure 2a), with the blocks in pairs 1, 2, 4-7, 9-11 and X chromosome being G+C-rich, while the rest of the heterochromatin was neutral for CMA_3_ or DAPI fluorochromes. The blocks in pairs 4 and 7 occurred in only one of the homologues. In pairs 3 and 5, terminal CMA_3_^+^ blocks were also noticed, being heteromorphic for pair 3 (Figure 2b). This pattern of C-positive pericentromeric blocks associated with CMA_3_^+^ heterochromatic blocks and/or heterochromatin without base specificity (A+T or G+C) observed for the A chromosomes of *Eumastusia koebelei koebelei* is similar to other Leptysminae species, such as *Cornops aquaticum* (Bruner, 1906), *Stenopola dorsalis*, *Stenacris xanthochlora* (Marschall, 1836), *Tucayaca parvula* Roberts, 1977 and *Belosacris coccineipes* (Bruner, 1906), as well as in other species of Acrididae (Hewitt 1979, King and John 1980, Loreto and de Souza 2000, Rocha et al. 2004). In the two B chromosomes, the heterochromatin was distributed along their entire length (Figure 2d), and in the pericentromeric region a remarkable CMA^3+^ block was noticed. This CMA_3_^+^ area appeared as a conspicuous block in metaphase I while in initial meiosis (pachytene), due to less condensation, dispersed dots were always observed side by side (Figure 2e, f) due to the association of the two B chromosomes. The shared CMA_3_^+^ block in both B chromosomes reinforces their similarity, and we could speculate that a G+C-rich repetitive DNA, such as satellite DNA, could be present in the centromere of these B chromosomes, facilitating their constant association. This situation could cause a centromeric division failure that could favor the occurrence of whole-arm translocations leading to the formation of an isochromosome, proposed as a hypothesis for B isochromosome origin in the grasshopper *Metaleptea brevicornis adspersa* (Johannson, 1763) (Grieco and Bidau 2000).

Another argument favoring the notion of repetitive DNA enrichment in the C-positive regions was confirmed through the use of the DOP-PCR fraction as a probe, which revealed strong signals in these areas (Figure 2c). This is also valid for the B chromosomes, which were completely labeled (Figure 2g). The enrichment of distinct classes of repetitive DNAs in B chromosomes is a common pattern and these sequences could be involved with B chromosome differentiation and evolution (Houben et al. 2014, Banaei-Moghaddam et al. 2015). Considering that the DOP-PCR probe was obtained from an individual without B chromosomes, the repetitive DNA amplified using this approach is from the A genome. The hybridization signals in the B chromosomes indicate that this element shares repetitive sequences with the A complement, suggesting an intraspecific origin for the B chromosome. An intraspecific origin for B chromosomes was also suggested for other grasshoppers using distinct chromosomal markers, such as *Abracris flavolineata* (Menezes-de-Carvalho et al. 2015) and *Locusta migratoria* (Linnaeus, 1758) (Teruel et al. 2010), as well as other animal groups. Our result is similar to reports for the beetle *Dichotomius geminatus* (Arrow, 1913) using as probe the *C_0_t*-1 DNA fraction that also isolates repetitive DNAs, such as the DOP-PCR, indicating the sharing of sequences between the B chromosome and the A complement (Cabral-de-Mello et al. 2011b). Although we suggest an intraspecific origin for the B chromosome in *Eumastusia koebelei koebelei*, it is impossible to determine if this event is related either to autosomes or the X chromosome, because both presented signals for the DOP-PCR probe and CMA_3_^+^ blocks. It is also impossible to define the specific type of shared sequence, as the DOP-PCR probe is anonymous.

FISH with the telomeric probe revealed terminal signals in all chromosomes, including the B chromosome (Figure 2i). This result was expected considering that the karyotype of *Eumastusia koebelei koebelei* does not experienced gross chromosomal rearrangements observed in other Leptysminae, such as *Stenopola pallida*, *Tetrataenia surinama* and *Leptysma argentina*, bearing in mind the ancestral karyotype for grasshoppers (Mesa et al. 1982, Bidau and Hasson 1984). For the B chromosome, this probe confirmed that one individual harbored two B chromosomes (Figure 2i, insert) instead of one large biarmed chromosome, as suggested by conventional analysis. The mapping of multigene families revealed one pair of clusters on the same bivalent for 18S rDNA, proximally in pair 9. For U2 snDNA, four clusters on two bivalents were noticed, with interstitial placement in pair 1 and 8 in decreasing order of size (Figure 2h, i). This multigene family and the U1 snDNA located in pair 3 (Anjos et al. 2015) were not observed in the B chromosomes (Figure 2 h, i). It is more parsimonious to consider that chromosomes 1, 3, 8 and 9 were not involved in the origin of B chromosomes, but it could not be completely ruled out. Alternatively, these sequences could be lost during B chromosome differentiation, or the origin of the B chromosome did not involve the regions containing these sequences.

## Conflict of interest

The authors declare that they have no conflict of interest.
